# Genetic variation in 9p21 is associated with fasting insulin in women but not men

**DOI:** 10.1371/journal.pone.0202365

**Published:** 2018-08-23

**Authors:** Sara Mahdavi, David J. A. Jenkins, Ahmed El-Sohemy

**Affiliations:** 1 Department of Nutritional Sciences, Faculty of Medicine, University of Toronto, Toronto, Ontario, Canada; 2 Li Ka Shing Knowledge Institute, Risk Factor Modification Centre and Division of Endocrinology and Metabolism, St. Michael's Hospital, St. Michael's Health Centre, Toronto, Ontario, Canada; Medizinische Universitat Innsbruck, AUSTRIA

## Abstract

**Background:**

Single nucleotide polymorphisms (SNPs) in the 9p21 region have been associated with cardiovascular disease (CVD), but previous studies have focussed on older populations. The objective of this study was to determine the association between 9p21 genotypes and biomarkers of CVD risk in young adults from different ethnocultural groups.

**Methods:**

Subjects were 1,626 participants aged 20–29 years from the Toronto Nutrigenomics and Health Study. Fasting blood was collected to measure glucose, insulin, c-reactive protein and serum lipids, as well as to isolate DNA for genotyping subjects for five SNPs in 9p21. Analyses were conducted for the entire population and separately for women (n = 1,109), men (n = 517), Caucasians (n = 771), East Asians (n = 561) South Asians (n = 175) and Others (n = 119). ANOVA and ANCOVA were used to examine if 9p21 genotypes were associated with biomarkers of CVD risk.

**Results:**

In the entire group, the risk alleles of rs10757278 and rs2383206 were associated with higher mean insulin (p = 0.01). Risk alleles for rs4977574, rs10757278, rs2383206, rs1333049 and rs10757274 were associated with higher serum insulin in women (p = 0.008, p = 0.008, p = 0.0003, p = 0.002, and p = 0.001, respectively), but not in men (p = 0.41, p = 0.13, p = 0.31, p = 0.34, and 0.35, respectively). The association between 9p21 and insulin remained significant among women not taking hormonal contraceptives (HC), but was not significant among women taking HCs.

**Conclusion:**

Our findings suggest that 9p21 genotypes may play a role in the development of insulin resistance in early adulthood among women, but not men, and the effects appear to be attenuated by HC use.

## Introduction

Cardiovascular disease (CVD) is a leading cause of mortality in Canada and worldwide [1] and its pathogenesis involves both environmental and genetic factors [2–4]. CVD risk is often associated with a cluster of intermediate phenotypes including glucose intolerance, dyslipidemia, hypertension and abdominal obesity, collectively referred to as markers of cardiometabolic disease (CMD), which are predictive of higher rates of CVD events later in life. Genetic factors play an important role in CVD events as demonstrated by several large-scale genome-wide association studies (GWAS) [5–7]. An 8-kilobase interval in the 9p21 region contains single nucleotide polymorphisms (SNPs) that have been associated with an increased risk of CVD in at least six different populations involving more than 23,000 participants [5,8]. These variants were in linkage disequilibrium (LD) and now have been shown to span a 58-kb region that has multiple neighboring genes (*CDKN2A*, *CDNK2B*, and *MTAP*), without annotating to any single protein sequence [5,8,9]. SNPs in this region have also been associated with type 2 diabetes mellitus (T2DM) and insulin sensitivity [10], although the evidence is not as strong as those seen for CVD. Risk alleles of 9p21 are common in most populations and an estimated 50% of Caucasians carry at least one copy with an estimated 30% increased risk of CVD compared to non-carriers [6]. Four of the most frequently reported 9p21 SNPs associated with CVD (rs10757274, rs10757278, rs4977574 and rs2383206) have an A>G substitution while another commonly reported SNP (rs1333049) has a G>C substitution [7,11–14]. CVD risk increases with each copy of 9p21 risk alleles when compared to those who are homozygous for the ancestral alleles [11]. The risk of CVD associated with 9p21 variants is even stronger in those 55 years and younger. In a meta-analysis of genetic risk factors for CVD [15], those who are heterozygous for the 9p21 risk allele have a 25% increased risk, while those who are homozygous for the risk allele have a 45% increased risk. These associated risks were greater among those diagnosed with CVD before 55 years of age compared to those diagnosed before 75 years of age, suggesting that 9p21 plays a bigger role in earlier cases of CVD where environmental risk factors might not have had enough time to impact the development of CVD. Given the long lag times between exposure to environmental risks and CVD outcomes, emphasis on preventive approaches to modify CVD risk at an earlier age may be more advantageous than treatment later in life [16–18], however, most studies are conducted in older, diseased adults. Although CVD is often diagnosed after an acute ischemic event in middle or late-life, its pathogenesis starts decades earlier [19], when clinical symptoms of CVD may be absent according to traditional markers.

In a landmark autopsy study of children, youth and young adults (ages 15 to 36) who had died of trauma (motor vehicle accidents, gun violence, other accidental injuries), evidence of atherosclerosis was found as early as the second decade of life [20]. Atherosclerotic lesions became progressively more prominent with each decade of life and with the presence of each additional modifiable CVD risk factor [20]. A recent study of factors differentiating biological versus chronological aging processes in a cohort of 26–38 year-olds showed that several factors, including genetics and diet determine rate of biological aging and susceptibility to chronic disease [21]. Similarly, both diet and genetics likely play a role in early development of CVD.

We aimed to test the hypothesis that 9p21 risk variants are associated with biomarkers of CVD risk in a population of young adults from different ethnocultural groups.

## Methods

### i) Study population

Participants were from the Toronto Nutrigenomics and Health Study (TNHS), which has been described elsewhere [22]. In brief, the TNHS is a cross-sectional study that aims to explore the link between diet, genes and biomarkers of chronic disease and was approved by the University of Toronto Research Ethics Board. Study participants were aged 20–29 years from various ethno-cultural backgrounds and were recruited from the University of Toronto campus between 2004 and 2010 who signed a written, informed consent approved by the University of Toronto Research Ethics Board (REB) to participate in the study. Anthropometric measurements including height, weight, waist circumference, and blood pressure were recorded according to standard procedures [22]. Subjects provided a fasting blood sample for DNA isolation and plasma was separated for measuring biomarkers of CVD risk including blood lipids, inflammatory markers and insulin.

### ii) DNA analysis and ethnocultural determination

DNA was extracted from whole blood samples using standard procedures and analyzed for the following SNPs in 9p21: rs2383206, rs10757274, rs10757278 and rs1333049. These SNPs have been shown most consistently to be associated with CVD risk in several populations and ethnicities globally [23]. Genotyping of 9p21 SNPs was completed at the Clinical Genomics Centre in the Princess Margaret Hospital, University Health Network, using the iPLEX Gold assay with mass spectrometry-based detection (Sequenom MassARRAY platform; Sequenom Inc) for all subjects. Ethnocultural status was determined by asking subjects in an open-ended format to self-report the ethnocultural group(s) they identify with. Subjects were then categorized into the three most commonly reported ethnocultural groups based on self reports and all others into a separate group. Caucasians included those who considered themselves European, Middle Eastern, or White-Hispanic. East Asians included Chinese, Japanese, Koreans, Filipinos, Vietnamese, Thai, and Cambodians. South Asians consisted of Bangladeshi, Indians, Pakistani, and Sri Lankans. The Others category included individuals who reported belonging to 2 or more ethnocultural groups not included in the same category, as well as Aboriginal Canadians, Africans or Afro-Caribbean.

### iii) Statistical analysis

Statistical Analysis Software v.9.4 (SAS Institute Inc, Cary, NC) was used for all analyses. Subjects with missing information on ethnicity, genotype or biomarkers of interest as well as any subjects who broke their 12-hour fast before blood sample collection were excluded. A total of 1,626 subjects with complete data were included in the analyses. Genotypes for 9p21 were examined for Hardy-Weinberg equilibrium. Chi-square test was used to assess 9p21 risk allele frequency across different ethnocultural groups. The distributions of all continuous variables were tested for normality and were log-transformed as needed, however, untransformed means and spreads were reported to facilitate interpretation of the data. The α-error was set at 0.05, and p-values presented are two-sided. Initially, all analyses were unadjusted, and then adjusted for several covariates. Only those variables that were statistically significant in most models or materially altered the outcomes were retained in the model. The variables in each model were also tested for multicollinearity with tolerance level set at <0.4. No multicollinearity was detected amongst the variables selected for the final models. Using analysis of covariance (ANCOVA), differences in mean biomarker concentrations were examined across 9p21 genotypes, initially in the whole group. In sub-group analyses, groups were stratified by four ethnocultural groups, men and women as well as users and non-users of hormonal contraceptives (HC) in women. The final models included adjustments for sex, ethnocultural group, serum glucose, diastolic blood pressure, log body mass index, log waist circumference, and hormonal contraceptive use by women, unless the outcome measure was stratified by one of these variables (such as sex, hormonal contraceptives or ethnocultural groups), in which case it was not included in the adjustment for that model. Post-hoc pair-wise differences were analyzed using Tukey’s test. The Benjamini-Yekutieli (B-Y) procedure [= α/∑ (1/i), where i varies from one to the total number of tests conducted] was applied to adjust for multiple testing (adjusted p < 0.02, calculated for testing the primary hypothesis of the study with six biomarkers of CVD as separate predictors with α <0.05 for each test). The B-Y method was selected because it allows for potential dependence between tests [24], and many of the markers used here are biologically related. Adjustments were not made for analyses of five 9p21 SNPs as they were in linkage disequilibrium with each other.

## Results

Subject characteristics are shown in Table 1. All five SNPs were in LD (ie >0.8). When the analyses where stratified by sex and four self-identified ethnocultural groups, risk allele frequencies of the five SNPs in each group were similar to those reported elsewhere and ranged from 46–55%. The only exception to this was in the smallest and most heterogeneous groups of Others, that were from a mixed ethnocultural origin. In the Others group, the frequency of the risk alleles for rs10757274, rs10757278 and rs1333049 was lower (34–36%). East Asians also had a slightly lower frequency of the risk allele (G) in rs2383206 (46%), compared to the whole group, however, this prevalence was within the range of what has been reported in populations elsewhere [25].

**Table 1:** Subject characteristics.

	Women (n = 1109)	Men (n = 517)	P
Age (years)	22.5 ± 2.4	22.8 ± 2.4	0.08
Height (cm)	163.2 ± 6.5	175.9 ± 7.2	< 0.001
Weight (kg)	59.7 ± 10.7	73.1 ± 12.7	< 0.001
Body mass index (kg/m^2^)	22.4 ± 3.5	23.6 ± 3.5	< 0.001
Waist circumference* (cm)	71.3 ± 7.6	80.1 ± 9.1	< 0.001
Systolic Blood Pressure (mmHg)	109 ± 0	123 ± 1	<0.001
Diastolic Blood Pressure (mmHg)	68.4 ± 0.4	72.1 ± 0.8	<0.001
Genotype Frequency (% Risk Allele)			
rs10757278	47	49	
Ethnocultural group (%)			
Caucasian	48	48	0.84
East Asian	36	31	0.14
South Asian	9	14	0.005
Other	8	7	0.87

Values are mean ± standard error, p-values are for comparison between two sexes using unadjusted linear regression model. Chi-square test was used to test for differences between genotypes in categorical variables.

*Variables were log-transformed to normalize distribution before use in regression

Traditional biomarkers of CVD were not significantly different when compared between genotypes, except for fasting insulin (Table 2). Mean fasting insulin for the entire study population was 47.4 ± 0.9 pmol/L. Mean fasting insulin was 8% higher in those who are heterozygous for the risk allele (AG) in rs10757278 and 16% higher for those who are homozygous (GG) (p = 0.01).

**Table 2:** Traditional biomarkers of CVD risk by rs10757278 genotype and ethnocultural group.

Group	CVD Biomarker		Genotype		P
All (n = 1,626)		**AA**	**AG**	**GG**	
	Glucose (mmol/L)	4.77 ± 0.02	4.79 ± 0.01	4.79 ± 0.02	0.44
	Insulin* (pmol/L)	44.0 ± 1.6	47.7 ± 1.3	51.1 ± 2.1	**0.01**
	CRP* (mmol/L)	1.35 ± 0.14	1.21 ± 0.08	1.25 ± 0.13	0.87
	TG* (mmol/L)	0.98 ± 0.14	0.98 ± 0.08	0.99 ± 0.13	0.99
	HDL* (mmol/L)	1.55 ± 0.02	1.54 ± 0.01	1.52 ± 0.02	0.71
	LDL (mmol/L)	2.27 ± 0.03	2.30 ± 0.02	2.27 ± 0.03	0.63
Caucasians (n = 771)					
	Glucose (mmol/L)	4.74 ± 0.02	4.77 ± 0.03	4.74 ± 0.02	0.54
	Insulin* (pmol/L)	40.3 ± 1.5	45.9 ± 2.0	46.9 ± 3.0	0.12
	CRP* (mmol/L)	1.45 ± 0.19	1.44 ± 0.13	1.45 ± 0.19	0.14
	TG* (mmol/L)	1.00 ± 0.05	0.97 ± 0.03	1.00 ± 0.04	0.99
	HDL* (mmol/L)	1.56 ± 0.04	1.55 ± 0.01	1.52 ± 0.04	0.48
	LDL (mmol/L)	2.30 ± 0.01	2.24 ± 0.05	2.23 ± 0.06	0.63
East Asians (n = 561)					
	Glucose (mmol/L)	4.77 ± 0.04	4.78 ± 0.06	4.83 ± 0.04	0.40
	Insulin* (pmol/L)	39.6 ± 1.7	44.6 ± 1.6	47.5 ± 2.9	**0.04**
	CRP* (mmol/L)	0.99 ± 0.22	0.69 ± 0.12	0.81 ± 0.17	0.08
	TG* (mmol/L)	0.97 ± 0.08	0.93 ± 0.05	1.05 ± 0.08	0.37
	HDL* (mmol/L)	1.53 ± 0.03	1.60 ± 0.03	1.57 ± 0.08	0.14
	LDL (mmol/L)	2.23 ± 0.05	2.29 ± 0.06	2.31 ± 0.08	0.17
South Asians (n = 175)					
	Glucose (mmol/L)	4.87 ± 0.05	4.95 ± 0.07	4.85 ± 0.08	0.33
	Insulin* (pmol/L)	71.0 ± 12.0	61.2 ± 4.7	64.1 ± 6.0	0.63
	CRP* (mmol/L)	2.08 ± 0.70	1.69 ± 0.33	1.20 ± 0.38	0.33
	TG* (mmol/L)	0.99 ± 0.07	1.16 ± 0.11	0.78 ± 0.04	0.46
	HDL* (mmol/L)	1.49 ± 0.06	1.30 ± 0.04	1.38 ± 0.05	**0.03**
	LDL (mmol/L)	2.26 ± 0.10	2.57 ± 0.08	2.26 ± 0.06	**0.02**
Others (n = 119)					
	Glucose (mmol/L)	4.78 ± 0.05	4.79 ± 0.06	4.88 ± 0.16	0.64
	Insulin* (pmol/L)	50.5 ± 31.4	56.3 ± 4.9	79.6 ± 16.3	**0.02**
	CRP* (mmol/L)	1.44 ± 0.32	1.76 ± 0.45	2.44 ± 0.79	0.20
	TG* (mmol/L)	0.93 ± 0.06	0.97 ± 0.08	1.12 ± 0.12	0.39
	HDL* (mmol/L)	1.61 ± 0.06	1.49 ± 0.06	1.60 ± 0.09	0.45
	LDL (mmol/L)	2.26 ± 0.09	2.32 ± 0.11	2.41 ± 0.15	0.35
Women (n = 1109)					
	Glucose (mmol/L)	4.70 ± 0.02	4.74 ± 0.01	4.71 ± 0.02	0.37
	Insulin* (pmol/L)	43.4 ± 1.4	49.0 ± 1.6	55.7 ± 2.9	**<0.01**
	CRP* (mmol/L)	1.32 ± 0.15	1.35 ± 0.11	1.4 ± 0.18	0.91
	TG* (mmol/L)	0.94 ± 0.02	0.94 ± 0.02	0.98 ± 0.05	0.22
	HDL* (mmol/L)	1.64 ± 0.02	1.65 ± 0.02	1.63 ± 0.02	0.94
	LDL (mmol/L)	2.25 ± 0.03	2.27 ± 0.03	2.28 ± 0.04	0.81
Men (n = 517)					
	Glucose (mmol/L)	4.91 ± 0.03	4.92 ± 0.02	4.95 ± 0.04	0.74
	Insulin* (pmol/L)	45.5 ± 3.9	44.8 ± 1.9	42.4 ± 2.5	0.22
	CRP* (mmol/L)	1.43 ± 0.34	0.9 ± 0.1	0.96 ± 0.17	0.81
	TG* (mmol/L)	1.06 ± 0.07	1.05 ± 0.05	1.02 ± 0.05	0.92
	HDL* (mmol/L)	1.34 ± 0.02	1.29 ± 0.02	1.31 ± 0.03	0.17
	LDL (mmol/L)	2.29 ± 0.06	2.36 ± 0.04	2.24 ± 0.06	0.22

Values are mean ± standard error, p-values are for comparison between three genotypes using unadjusted linear regression model.

*Variables were log-transformed to normalize distribution for model building. CRP, C-reactive protein; HDL, high density lipoproteins; LDL, low density lipoproteins; TG, triglycerides.

Fasting insulin was then assessed across all five 9p21 SNPs and similar patterns of a stepwise increase in fasting insulin were seen with each additional risk allele for all five SNPs. However, only differences in genotypes of rs10757278 and rs2383206 were statistically significant. Mean fasting insulin for Caucasians was 44.6 ± 1.3 pmol/L, for East Asians 43.9 ± 1.1 pmol/L, for South Asians 63.4 ± 4.0 pmol/L, and for the Others 57.0 ± 3.7 pmol/L. Among those in the Others group, the presence of two risk alleles in rs10757278 was associated with approximately 30 pmol/L higher mean insulin when compared to no risk alleles (pairwise comparison, p = 0.031) (S1 Table).

When the study population was stratified by sex, the association between 9p21 genotype and fasting insulin was significant for women with all five SNPs (p-value between 0.008 to 0.0003) but not for men (S1 Table). Mean insulin for men was 44.4 ± 1.5 and for women 48.8 ± 1.1. Fasting serum insulin remained relatively constant across genotypes in men, but there was a 13% increase with one risk allele and a 28% increase with two risk alleles in women. Furthermore, there was a significant sex-genotype interaction for all five SNPs analyzed (Table 3). Despite the lower number of men than women, the study was well powered to detect at least one unit of change in mean insulin difference in men. However, effect size was much smaller among genotypes it is expected that the mean differences in men would not be statistically significant.

**Table 3:** Interaction of 9p21 SNPs with sex and ethnocultural groups.

9p21 SNP	p	p	p-interaction	p-interaction
	Ethnicity	Sex	Ethnicity x gene	Sex x gene
rs10757274	0.16	**<0.001**	0.89	**0.009**
rs10757278	0.11	**<0.001**	0.92	**0.004**
rs2383206	0.06	**<0.001**	0.82	**0.004**
rs1333049	0.18	**<0.001**	0.97	**0.012**
rs4977574	0.43	**<0.001**	0.68	**0.019**

p-values are for linear regression models adjusted for: ethnocultural group, sex, hormonal contraceptives in women, log-body mass index, diastolic blood pressure, log-waist circumference and plasma glucose

**Fig 1:**
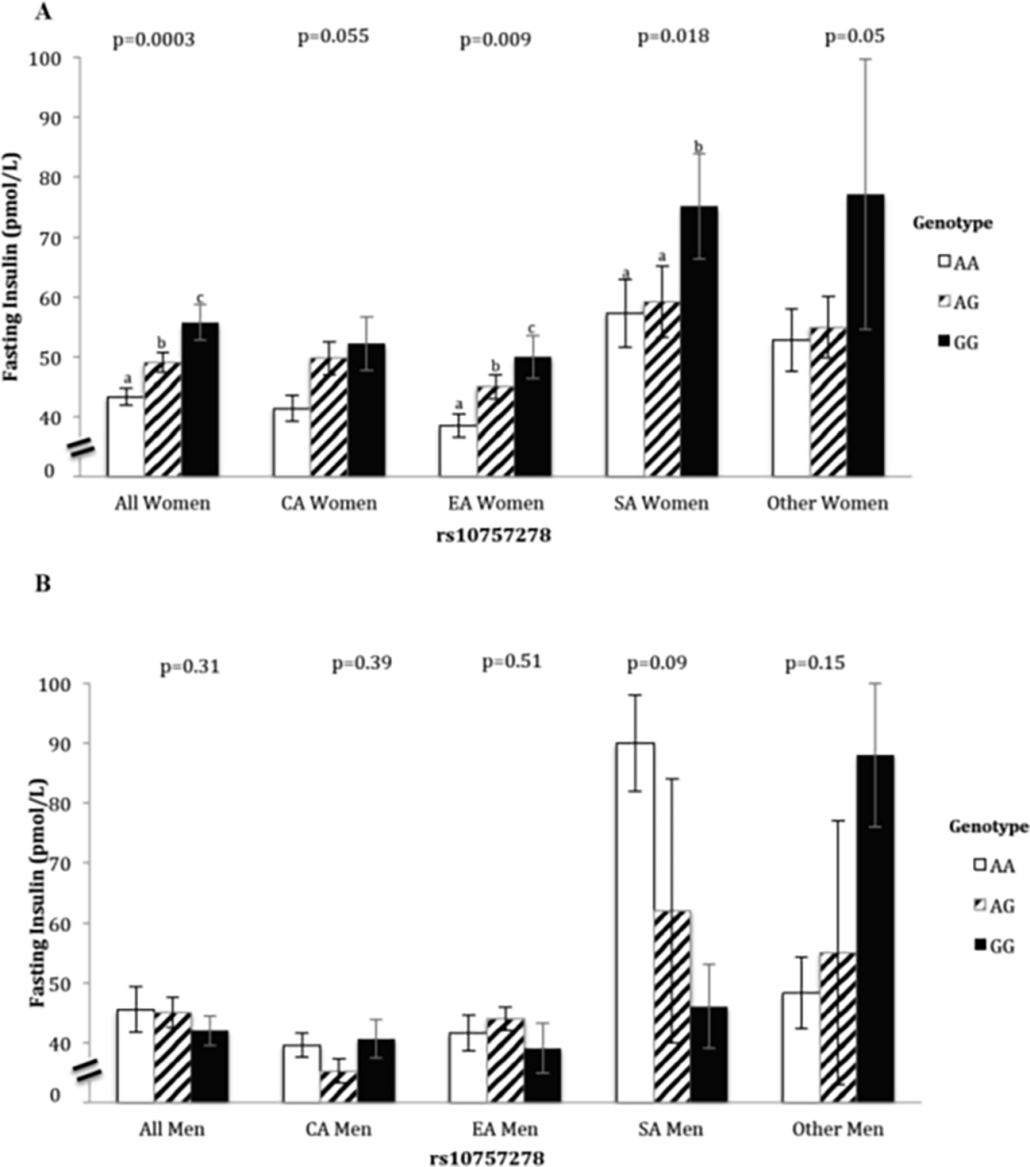
Fasting serum insulin by 9p21 genotype (rs10757278) in women (A) and men (B). *ANCOVA was adjusted for*: *log-body mass index*, *diastolic blood pressure*, *log-waist circumference and plasma glucose*. *Fasting Insulin (pmol/L) was log-transformed*, *but expressed as normal mean to facilitate interpretation*. *Different letters*, *a*, *b and c indicate statistically significant differences in insulin concentrations between genotypes (pairwise differences) within a group which were tested only if overall ANCOVA for the group was statistically significant*. *p-value at the top of each group is indicative of overall differences in each group for comparing all three genotypes within the group*. *CA; Caucasians*, *EA; East Asians*, *SA; South Asians*.

Despite variation in mean fasting insulin between ethnocultural groups among women, increasing fasting insulin was consistently associated with the presence of risk alleles in a stepwise manner across all groups. These associations were significance for East Asians and South Asians, but not for Caucasians and Others (Fig 1). Results of additional subgroup analyses are listed in S1 Table.

Among the women, 30% were on HCs and had higher mean fasting insulin levels than those not taking HCs (50.0 pmol/L ± 1.5 versus 48.3 pmol/L ± 1.4, p = 0.01, Table 4). When women were stratified according to HC use, the association between 9p21 genotype and fasting insulin remained statistically significant for some ethnocultural groups, but no associations were observed among women taking HCs for any ethnocultural group (Table 4). This was most striking in the Caucasians, the largest group, where mean fasting insulin in women who were not taking HCs was 1.5 times higher in those who were homozygous for the G allele compared to those who were homozygous for the A allele (p = 0.0002, Table 4). In the Caucasian HC users, on the other hand, no differences in fasting insulin means were observed when comparing the same genotypes (p = 0.93, Table 4).

**Table 4:** Fasting insulin associated with rs10757278 according to HC use in women.

Ethnocultural Group	AA	Genotype AG	GG	p
**All Women**				
No HC (n = 784)	41.7 ± 1.7	48.5 ± 2.1	56.5 ± 3.9	**0.0002**
On HC (n = 325)	47.5 ± 2.6	50.1 ± 2.1	53.6 ± 3.9	0.63
**Caucasians**				
No HC (n = 295)	37.7 ± 2.9	50.2 ± 4.3	53.2 ± 7.6	**0.02**
On HC (n = 223)	46.2 ± 2.9	49.1 ± 2.5	51.1 ± 4.5	0.93
**East Asians**				
No HC (n = 346)	37.4 ± 2.0	44.2 ± 2.2	47.8 ± 3.8	**0.01**
On HC (n = 52)	48.7 ± 6.1	50.2 ± 5.3	61.8 ± 10.2	0.31
**South Asians**				
No HC (n = 87)	53.8 ± 5.6	60.4 ± 7.1	77.1 ± 9.2	**0.02**
On HC (n = 18)	69.0 ± 15.9	54.4 ± 10.4	46.5 ± 21.9	0.41
**Others**				
No HC (n = 54)	40.3 ± 5.3	55.5 ± 6.3	60.3 ± 11.5	0.60
On HC (n = 28)	58.8 ± 6.9	54.6 ± 7.3	85.5 ± 33.2	0.08

ANCOVA was adjusted for: log-body mass index, diastolic blood pressure, log-waist circumference and plasma glucose. Fasting Insulin was log-transformed to normalize distribution, but expressed as normal mean to facilitate interpretation. HC, hormonal contraceptives.

In East Asians, similar findings were observed as those seen in Caucasians, where those taking HCs had a mean fasting insulin of 53.0 ± 4.1 pmol/L versus 43.1± 1.5 pmol/L for those not taking HCs (p = 0.007 for pairwise comparison). In this group of East Asian women, fasting insulin was only associated with genotype in those not using HCs (p = 0.02, Table 4). South Asian women and women from the Others group had a much higher mean concentration of fasting insulin (63.0 ± 4.1 pmol/L and 57.2 ± 4.1 pmol/L, respectively) even though the pattern of association was similar to all other ethnocultural groups (p = 0.018 and p = 0.05, Fig 1). The association between 9p21 risk alleles and fasting insulin was the same in women not on HC from all ethnocultural groups, despite significantly different mean fasting insulin in each ethnocultural group. Among the women who were not taking HCs, serum insulin was highest in South Asians (64.2 ± 4.5 pmol/L) compared to Caucasians (47.4 ± 2.8 pmol/L) and East Asians (43.1 ± 1.5 pmol/L) (p<0.001, all pairwise comparisons). Although the South Asian women not on HCs had the highest mean fasting insulin levels for all three genotypes, the mean differences among the genotypes were significantly different (p = 0.02, Table 4). All raw data collected and used for the purposes of the current manuscript are included in S2 Table.

## Discussion

Variations in 9p21 are the most robust genetic markers of CVD. We sought to examine the associations between 9p21 genotype with early biomarkers of CVD risk in over 1,600 young-adults. Other studies on 9p21 are almost exclusively conducted in older adults [26], where CVD risk confounders are likely present in addition to genetic risk of 9p21. Therefore, despite this region being the subject of many studies over the past 15 years, the biological mechanisms by which it is linked to CVD development are still largely unknown. To our knowledge, this study is the first to examine 9p21 genotypes and biomarkers of CVD risk in young adults in their early 20’s from different ethnocultural groups.

We found that risk alleles of five most commonly studied SNPs in the 9p21 region were associated with higher levels of fasting serum insulin in this cohort, with rs10757278 showing the strongest association. This suggests that pathways involving insulin might be involved in mechanisms by which the 9p21 region is linked with CVD. Higher fasting insulin levels are associated with numerous adverse risk factors in young adults, increasing the risk of atherosclerosis [27] and subsequent type 2 diabetes [28]. Even in nondiabetic individuals, hyperinsulinemia is associated with decreases in insulin-mediated glucose uptake [29], as well as with a number of clinical symptoms associated with insulin resistance [30,31]. Prolonged and untreated insulin resistance and hyperinsulinemia are known to be associated with hypertriglyceridemia [32] and low concentrations of high-density lipoprotein cholesterol [32,33], hypertension [34], and coronary artery calcification [35], which are all risk factors for CVD. Hyperinsulinemia has also independently been associated with ischemic heart disease [31].

The synergism of risk alleles in the 9p21 locus and hyperglycemia have also been demonstrated in coronary artery disease (CAD) development and accelerated CVD mortality in those with type 2 diabetes [36]; where those homozygous for the risk alleles had worse glycemic control and higher incidents of CAD than others. Although 9p21 does not contain annotated genes, rs10757278 is in high linkage disequilibrium with two known genes CDKN2A and CDKN2B. These genes code for three known proteins p15^INK4b^, p16 ^INK4b^ and ARF, that have been linked to the presence of hereditary atherosclerosis [37,38] where they inhibit cyclin-dependent kinases controlling cell proliferation and apoptosis in endothelial layer and vascular smooth muscle, among other tissue. One study found that antisense noncoding RNA in the INK4 locus (ANRIL) expression were significantly higher in carriers of the 9p21 risk alleles, with expression directly correlating with atherosclerotic severity [39]. The findings in our study suggest that insulin-dependent pathways might be one of the earliest signs of metabolic dysregulation, linking 9p21 risk alleles to CVD risk factors, evident in early adulthood.

We observed a significant genotype-sex interaction on fasting insulin, which has not been previously reported. The associations were observed in women, but not in men. Similarly, in the ADVANCE study, where genotypic odds ratio (OR) was stratified by sex, a trend towards higher ORs in women rather than men was observed [40]. The authors however concluded that this was likely due to admix of genotype present in the population, since the risk allele frequency differed between men and women in that cohort [40]. More recently, GG genotype of rs10757278 was also shown to be significantly and independently associated with carotid plaque in women only [41]. Insulin resistance, hormonal glucose intolerance and type 2 diabetes increase the risk of CVD and mortality, however women are more affected as it increases the risk of CVD death by about four times in women verses two-fold in men [42]. In other studies, women have been identified as being distinctly different than men with regard to insulin action, susceptibility to develop insulin resistance, and response to stimuli that are known to enhance or impair sensitivity to the effects of insulin [43,44]. In the present study, we report a sex-specific genetic association between 9p21 and fasting insulin that might contribute to our understanding of how 9p21 impacts the development of CVD.

We also demonstrated varying magnitudes of these associations between the four ethnocultural groups of women (Fig 1). In the present study, South Asian and East Asian women had the largest differences in their mean fasting insulin between genotypes (p = 0.018 and p = 0.009, respectively), and the direction of associations was consistent amongst all groups. These findings of differences in ethnocultural groups are in agreement with others where South Asians have been shown to have an increased risk of developing CVD compared with Caucasian populations [45], while East Asians have a lower incidence of CVD compared to Caucasians [46]. Furthermore, the high prevalence of insulin resistance and type 2 diabetes in South Asians has been attributed as a major cause for their elevated CVD risk at a younger age [45,47] compared to other ethnocultural groups. Similarly, in the present study, the mean insulin for South Asians was significantly higher than mean serum insulin in all other ethnocultural groups, suggesting additional factors might contribute to the observed higher insulin levels this population. A novel aspect of the present study is the young age of the cohort (22.7 ± 2.4 years), which highlights the possibility of very early onset of these ethnocultural differences in disease risk. Furthermore, genotype associations remained consistent in women, irrespective of ethnocultural group. In other studies, although variants at the 9p21 locus were previously associated with risk of myocardial infarction in South Asians, the strength of the associations were weaker in South Asians than in Europeans [48]. However, this weaker association might be explained by the fact that the analyses of that study were not stratified by sex. In the present study, similar patterns were observed when men and women from the South Asian group were analyzed together versus the marked differences observed when comparing men and women separately in the entire cohort.

Additional analyses in the present study demonstrated that when women were grouped according to HC use, those on HCs had a significantly higher mean fasting insulin with no significant association between genotype and fasting insulin. In contrast, those who were not taking HCs had lower mean fasting insulin which was significantly associated with 9p21 genotype (Table 4). HC use has been associated with insulin resistance, manifested by reduced peripheral tissue insulin sensitivity [49], however this association has not been shown to be related to 9p21 genotype elsewhere. In the present study, Caucasian women formed the largest ethnocultural group, where the number of those on HC versus those without was similar (Table 4). When comparing mean fasting insulin according to genotype, a protective effect seems to be present in carriers of the A allele for rs10757278, whereas in those taking HCs this protection was not observed, despite having the same genotype and ethnocultural group.

Currently in clinical settings, biomarkers of CVD risk, including elevated CRP, and dyslipidemia determine intermediate phenotypic presentation of CVD and predict 10-year risk of CVD events. However, in the present study, similar to others [26], we found no meaningful associations between other traditional CVD biomarkers including lipid profile, BMI, or hypertension suggesting that insulin-related associations observed might be either independent of those involved in cardiometabolic disease or the earliest dysregulations seen in the cascade of phenotypic events. Although the mechanisms by which 9p21 SNPs influence CVD risk are not well defined, a link between CVD genetic susceptibility and the response to inflammatory signaling has been reported. No clear relationship between inflammatory markers and 9p21 genotype was observed in the present study, and this could be due to the young age of the study participants and the observational nature of the study design. However, others have reported long-distance interactions of CDKN2A/B, the MTAP gene, and interval downstream of IFNA21human vascular endothelial cells [50]. Interferon-γ (IFNγ) activation seems to affect the structure of chromatin, therefore, transcriptional regulation in the 9p21 locus, including STAT1-binding, likely plays a role as a long-range enhancer, altering expression of neighbouring genes [50]. One study identified 33 enhancers in the 9p21 region, with rs10757278 located in one of these enhancers, where the presence of the risk allele (G), disrupted a binding site for STAT1 [50]. The link between this disturbance and insulin resistance was demonstrated by another study where IFNγ was shown to induce insulin resistance in mature human adipocytes [51]. In the present study, we demonstrated that 9p21 risk alleles were associated with higher serum insulin levels, particularly with rs10757278. This finding may be explained by a possible mechanism whereby the A>G substitution in rs10757278 disrupts STAT1 binding site and modifies IFNA21 gene expression. IFNγ then possibly induces insulin insensitivity seen as higher insulin to be an early marker of endothelial dysfunction and increased susceptibility to CVD later in life. However, given the cross-sectional nature of the present study, causality cannot be determined from these results.

Early detection of CVD is necessary to improve health outcomes, however, most studies in this area have been in older populations. Currently, biomarkers of CVD risk are used as a tool for early intervention in clinical settings, however, these interventions are not personalized to individual risk factors such as genetic susceptibility, sex, ethnicity or environmental factors. Here we demonstrate that biomarkers of CVD risk that signify early metabolic disturbances related to CVD are present in early adulthood, and differ by genetic risk factors, sex and hormonal contraceptive use in women. This approach can potentially have an added value to the traditional biomarkers used to treat or prevent CVD in high risk populations. In one study, adding the 9p21 allele to traditional risk factors was associated with improved predictably of CVD hazard ratio of incident coronary heart disease of 1.2 per allele (p<0.000003) [52]. The currently study provides further support in better defining at risk groups that might have otherwise been missed using traditional markers only.

Chromosome 9p21.3 also encompasses variants strongly associated with T2DM and spatially arranged in a very tight genomic region adjacent, but distinct from the CVD-associated SNPs [53]. The LD between the respective lead SNPs of T2DM and CVD blocks have been very low (r^2^ <0.009) to modest (r^2^ = 0.35) for a secondary T2DM SNP near the CVD region (lead CVD SNP rs1333049 and T2D SNPs rs944801 and rs10811661) [53]. Hence the two regions have a low chance of mixing together during recombination, suggesting a distinct pattern of inheritance and rendering the associations observed with fasting insulin in the current study novel.

In summary, the present study is the first to demonstrate that common risk variants in the 9p21 region are associated with elevated fasting insulin in young adult women, but not in men, and not in women taking HCs. The role of 9p21 in insulin signaling and glucose metabolism in young women and its possible links to CVD risk warrants further investigation.

## Supporting information

S1 Table Mean fasting insulin according to risk alleles by sex and ethnocultural group. (DOCX)

S2 Table Study data used for all manuscript analyses. (XLSX)
