# Supplementary material for: Genetic variation in 9p21 is associated with fasting insulin in women but not men
Source: PLoS One. 2018 Aug 23;13(8):e0202365. doi: 10.1371/journal.pone.0202365 (PMC6107190; doi:10.1371/journal.pone.0202365)
Supplement: S1 Table — (DOCX) [file pone.0202365.s001.docx]

**S1Table:** Mean fasting insulin according to risk alleles by sex and ethnocultural group

| **Group** | **9p21 SNP** | **Risk allele** | **Mean Fasting Insulin** | | | **P^U^** | **P^A^** |
| --- | --- | --- | --- | --- | --- | --- | --- |
| All |  |  | **Homozygous** | **Heterozygous** | **Homozygous** |  |  |
| N=1604 | rs10757274 | G | 45.5 ± 1.6 | 47.5 ± 1.3 | 49.9 ± 1.9 | 0.17 | 0.11 |
| N=1615 | rs10757278 | G | 44.0 ± 1.6 | 47.7 ± 1.3 | 51.1 ± 2.1 | **0.01** | **0.03** |
| N=1616 | rs2383206 | G | 44.5 ± 1.8 | 47.2 ± 1.3 | 50.8 ± 1.9 | **0.02** | **0.04** |
| N=1616 | rs1333049* | C | 44.4 ± 1.6 | 47.6 ± 1.3 | 50.7 ± 2.1 | 0.05 | 0.05 |
| Caucasians |  |  |  |  |  |  |  |
| N=761 | rs10757274 | G | 41.8 ± 1.7 | 46.2 ± 2.2 | 44.8 ± 2.1 | 0.57 | 0.48 |
| N=764 | rs10757278 | G | 40.3 ± 1.6 | 46.0 ± 2.1 | 46.9 ± 3.0 | 0.12 | 0.29 |
| N=765 | rs2383206 | G | 40.8 ± 1.7 | 45.4 ± 2.2 | 46.4 ± 2.3 | 0.28 | 0.18 |
| N=765 | rs1333049* | C | 46.7 ± 3.0 | 45.9 ± 2.1 | 40.7 ± 1.6 | 0.17 | 0.43 |
| East Asians |  |  |  |  |  |  |  |
| N=554 | rs10757274 | G | 41.5 ± 2.1 | 43.8 ± 1.5 | 47.4 ± 2.9 | 0.19 | 0.34 |
| N=559 | rs10757278 | G | 39.6 ± 1.7 | 44.6 ± 1.6 | 47.5 ± 2.9 | 0.08 | 0.14 |
| N=558 | rs2383206 | G | 41.2 ± 2.1 | 43.9 ± 1.5 | 47.5 ± 2.9 | 0.12 | 0.20 |
| N=558 | rs1333049* | C | 39.7 ± 1.7 | 44.7 ± 1.6 | 47.0 ± 2.9 | 0.12 | 0.19 |
| South Asians |  |  |  |  |  |  |  |
| N=172 | rs10757274 | G | 70.3 ± 11.9 | 62.5 ± 4.9 | 63.7 ± 6.1 | 0.69 | 0.06 |
| N=175 | rs10757278 | G | 71.0 ± 12.1 | 61.2 ± 4.7 | 64.2 ± 6.0 | 0.66 | 0.05 |
| N=175 | rs2383206 | G | 70.2 ± 12.9 | 62.2 ± 4.9 | 63.5 ± 5.6 | 0.87 | 0.79 |
| N=175 | rs1333049* | C | 70.0 ± 11.5 | 61.5 ± 4.7 | 64.1 ± 6.2 | 0.73 | 0.07 |
| Others |  |  |  |  |  |  |  |
| N=118 | rs10757274 | G | 52.0 ± 4.4 | 54.8 ± 4.5 | 80.7 ± 18.5 | 0.27 | 0.83 |
| N=118 | rs10757278 | G | 50.5 ± 4.2 | 56.3 ± 4.9 | 79.1 ± 16.3 | 0.10 | 0.66 |
| N=118 | rs2383206 | G | 51.1 ± 6.8 | 53.2 ± 3.7 | 76.5 ± 13.5 | 0.09 | 0.58 |
| N=118 | rs1333049* | C | 52.0 ± 4.5 | 54.6 ± 4.6 | 77.9 ± 16.4 | 0.19 | 0.54 |
| Women |  |  |  |  |  |  |  |
| N=1093 | rs10757274 | G | 44.8 ± 1.6 | 49.4 ± 1.7 | 53.5 ± 2.6 | **0.009** | **0.005** |
| N=1100 | rs10757278 | G | 43.4 ± 1.4 | 49.0 ± 1.6 | 55.7 ± 2.9 | **0.0004** | **0.0003** |
| N=1102 | rs2383206 | G | 43.5 ± 1.7 | 48.6 ± 1.6 | 54.9 ± 2.5 | **0.0002** | **0.0004** |
| N=1101 | rs1333049* | C | 43.8 ± 1.5 | 48.9 ± 1.6 | 55.3 ± 2.9 | **0.002** | **0.001** |
| Men |  |  |  |  |  |  |  |
| N=512 | rs10757274 | G | 47.1 ± 4.0 | 43.5 ± 1.9 | 43.1 ± 2.5 | 0.49 | 0.13 |
| N=515 | rs10757278 | G | 45.5 ± 3.9 | 44.8 ± 1.9 | 42.4 ± 2.5 | 0.80 | 0.31 |
| N=515 | rs2383206 | G | 46.6 ± 4.3 | 44.0 ± 2.0 | 43.3 ± 2.3 | 0.62 | 0.34 |
| N=515 | rs1333049* | C | 45.7 ± 3.8 | 45.0 ± 1.9 | 41.8± 2.5 | 0.63 | 0.35 |

*Values are mean fasting insulin ± standard error listed in order of increasing genetic risk alleles; P^U^, p-values are for comparison between three genotypes using unadjusted linear regression models where insulin was log-transformed to normalize its distribution; P^A^**, p-values are linear regression models adjusted for: ethnocultural group, sex, hormonal contraceptives in women, log-body mass index, diastolic blood pressure, log-waist circumference and plasma glucose where applicable. * In all SNPs an A<G substitution was noted as the risk allele however for rs1333049 a G<C substitution signified risk allele as C, therefore the order for the genotype of this SNP is listed as GG, GC, CC; A, adenine; C; cytosine g, Guanine; SNP, single nucleotide polymorphism*
